# Spontaneous internal desynchronization of locomotor activity and body temperature rhythms from plasma melatonin rhythm in rats exposed to constant dim light

**DOI:** 10.1186/1740-3391-4-6

**Published:** 2006-04-04

**Authors:** Jacopo Aguzzi, Nicole M Bullock, Gianluca Tosini

**Affiliations:** 1 Neuroscience Institute and NSF Center for Behavioral Neuroscience, Morehouse School of Medicine, Atlanta, GA 30310-1495, USA; 2 Instituto de Ciencias del Mar (ICM-CSIC), Paseo Maritimo de la Barcelonesa 37-49; 08003, Barcelona, Spain

## Abstract

**Background:**

We have recently reported that spontaneous internal desynchronization between the locomotor activity rhythm and the melatonin rhythm may occur in rats (30% of tested animals) when they are maintained in constant dim red light (LL_dim_) for 60 days. Previous work has also shown that melatonin plays an important role in the modulation of the circadian rhythms of running wheel activity (R_w_) and body temperature (T_b_). The aim of the present study was to investigate the effect that desynchronization of the melatonin rhythm may have on the coupling and expression of circadian rhythms in R_w _and T_b_.

**Methods:**

Rats were maintained in a temperature controlled (23–24°C) ventilated lightproof room under LL_dim _(red dim light 1 μW/cm^2 ^[5 Lux], lower wavelength cutoff at 640 nm). Animals were individually housed in cages equipped with a running wheel and a magnetic sensor system to detect wheel rotation; T_b _was monitored by telemetry. T_b _and R_w _data were recorded in 5-min bins and saved on disk. For each animal, we determined the mesor and the amplitude of the R_w _and T_b _rhythm using waveform analysis on 7-day segments of the data. After sixty days of LL_dim _exposure, blood samples (80–100 μM) were collected every 4 hours over a 24-hrs period from the tail artery, and serum melatonin levels were measured by radioimmunoassay.

**Results:**

Twenty-one animals showed clear circadian rhythms R_w _and T_b_, whereas one animal was arrhythmic. R_w _and T_b _rhythms were always strictly associated and we did not observe desynchronization between these two rhythms. Plasma melatonin levels showed marked variations among individuals in the peak levels and in the night-to-day ratio. In six rats, the night-to-day ratio was less than 2, whereas in the rat that showed arrhythmicity in R_w _and T_b _melatonin levels were high and rhythmic with a large night-to-day ratio. In seven animals, serum melatonin levels peaked during the subjective day (from CT0 to CT8), thus suggesting that in these animals the circadian rhythm of serum melatonin desynchronized from the circadian rhythms of R_w _and T_b_. No significant correlation was observed between the amplitude (or the levels) of the melatonin profile and the amplitude and mesor of the R_w _and T_b _rhythms.

**Conclusion:**

Our data indicate that the free-running periods (τ) and the amplitude of R_w _and T_b _were not different between desynchronized and non-desynchronized rats, thus suggesting that the circadian rhythm of serum melatonin plays a marginal role in the regulation of the R_w _and T_b _rhythms. The present study also supports the notion that in the rat the circadian rhythms of locomotor activity and body temperature are controlled by a single circadian pacemaker.

## Introduction

Circadian rhythms in physiology and behavior have been described in a wide variety of organisms ranging from bacteria to humans. These rhythms are driven by circadian pacemakers that are capable of generating oscillations with a periodicity close to 24 hours. Several studies have shown that in many organisms the rhythms of locomotor activity and body temperature are under circadian control, and, although the level of activity may influence the body temperature, the circadian rhythm of body temperature is not a mere consequence of the circadian rhythm of locomotor activity (Review in [1]).

In mammals, the principal circadian pacemaker is located in the suprachiasmatic nuclei (SCN), bilateral clusters of neurons in the anterior hypothalamus. This circadian pacemaker regulates the different rhythms present in the body in order that the different circadian rhythms remain synchronized and maintain a stable phase relationships among themselves [2]. However, it must be noted that desynchronization among circadian rhythms may occur under specific experimental conditions. For example, spontaneous internal desynchronization between the body temperature (T_b_) and locomotor activity rhythms has been observed in reptiles [3] and in the squirrel monkey [4]. In the owl monkey, internal desynchronization between circadian activity and the feeding patterns has also been reported [5]. A recent investigation has shown that exposure to dim illumination may uncouple several circadian rhythms (e.g., sleep, body temperature, locomotor activity and drinking) in the rat [6]. Internal desynchronization has been also reported in humans [7,8], and it is believed to be the cause of several pathologies [9,10].

Previous studies have shown that melatonin is an important component of the mammalian circadian timing system. Exogenous administration of melatonin can entrain the circadian locomotor activity [11-13], and T_b _is affected by melatonin levels [12,14]. We have recently reported that desynchronization of the running wheel activity (R_w_) rhythm from serum melatonin may occur in rats exposed to constant dim red light (LL_dim_, [15]). The aim of the present study was to further expand this finding by investigating the effects that such a desynchronization may produce on the coupling and the expression of circadian rhythms of R_w _and T_b_.

## Materials and methods

Twenty-two male Wistar rats (Charles River Laboratory, Wilmington, MA), eight weeks old at the start of experiment, were used in this study. For T_b _recording, rats were implanted under anesthesia (ketamine/xylazine, 50 mg/Kg) with a transmitter (XM-FM, Mini-Mitter Inc., Bend, OR). After surgery, animals were immediately returned to their respective cages and allowed to recover for three days. Then, rats were transferred to a temperature controlled (23–24°C) ventilated lightproof room under LL_dim _(red dim light 1 μW/cm^2 ^[5 Lux]). Light was provided by a special fluorescent fixture (Litho light # 2, lower wavelength cutoff at 640 nm). Rats were individually housed in cages equipped with a running wheel and a magnetic sensor system to detect wheel rotation (Mini-Mitter Inc. Bend, OR). T_b _and R_w _data were recorded in 5-min bins and saved on disk using specific software (*Tau*, Mini-Mitters Inc.). For each animal, we determined the mesor and the amplitude of the R_w _and T_b _rhythm using waveform analysis on 7-day segments of the data.

After 60 days of LL_dim _exposure, blood samples (80–100 μM) were collected every 4 hours over a 24-hrs period from the tail artery in heparinized tubes. For each animal, the time of sampling was determined based upon each animal's locomotor activity rhythm. CT12 was defined as the time at which an animal began its daily bout of wheel running activity. All other circadian times were calculated relative to CT12. Melatonin was extracted from the serum (50 μM) using chloroform and then melatonin levels were measured by radioimmunoassay using a commercially available kit (ALPCO Diagnostics, Salem, NH). The sensitivity of the assay was 0.2 pg/ml. Intra-Assay variability was 9% and the inter-Assay was 13% (see [15] for more details).

Analysis of the R_w _and T_b _rhythms were performed on a 7-day segment of the data (i.e., from day 53 to day 60) using the Clock Lab software (Actimetrics, Evanston, IL). All the experiments reported here conformed to the guidelines outlined in the Guide for the Care and Use of Laboratory Animals from the U.S. Department of Health and Human Services and were approved by the Morehouse School of Medicine Institutional Animal Care and Use Committee.

## Results

**Figure 1:**
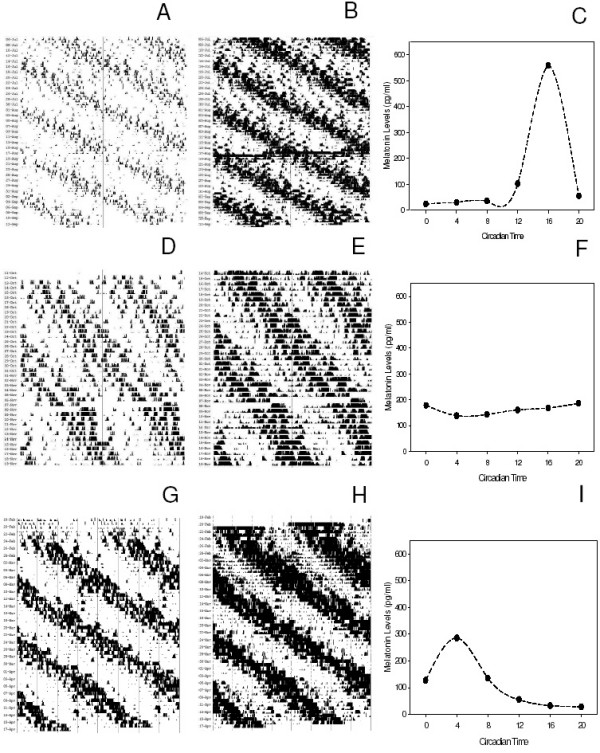
**Representative actograms of R_w_, T_b _and serum melatonin profile**. A, B, C: a synchronized animal (rat # 1 in Table 1); D, E. F: an animal with a damped melatonin rhythm (rat # 5 in Table 1); G, H, I: a desynchronized animal (rat # 21 in Table 1). Plots D and E show only the last 40 days of the experiment.

Out of twenty-two animals, twenty-one showed circadian rhythms in R_w _and T_b _for the entire duration of the experiment, whereas one rat became arrhythmic after 30 days of exposure to LL_dim _(see Figure 1 and Table 1). No desynchronization between the circadian rhythm of R_w _and T_b _and no significant changes in the τ of R_w _and T_b _rhythms were detected during the 60 day period (t-tests, P > 0.1 in all cases, Figure 1).

**Table 1:** Circadian parameters for R_w _and T_b_(mean ± SEM) and serum melatonin for each animal tested. Animals in which the circadian rhythm of serum melatonin was desynchronized from R_w _and T_b_, are indicated in bold. Animals in which the serum melatonin rhythm was damped are indicated in italic. (Amp. = amplitude)

	*Running Wheel*			*Body Temperature*			*Melatonin*	
	Mesor	Amp.	τ	Mesor	Amp.	τ	Range	Peak
Rat # 1	0.6 ± 0.2	19.3 ± 3.1	25.4	37.2 ± 0.1	2.1 ± 0.2	25.4	26–559.	16
**Rat # 2**	**0.7 ± 0.1**	**19.3 ± 3.1**	**25.1**	**36.8 ± 0.1**	**1.8 ± 0.2**	**25.0**	**12–122**	**4**
**Rat # 3**	**0.1 ± 0.1**	**5.6 ± 1.2**	**24.6**	**37.0 ± 0.1**	**1.9 ± 0.2**	**24.6**	**10–146**	**4**
Rat # 4		NS			NS		12–544	NA
*Rat # 5*	*6.6 ± 1.9*	*45.6 ± 2.1*	*25.1*	*37.6 ± 0.1*	*2.8 ± 0.2*	*25.2*	*105–204*	*16*
Rat # 6	2.6 ± 0.7	39.7 ± 5.0	24.3	37.5 ± 0.2	1.9 ± 0.2	24.3	38–306	16
*Rat # 7*	*3.4 ± 1.1*	*30.8 ± 3.1*	*24.4*	*36.9 ± 0.2*	*1.7 ± 0.1*	*24.4*	*109–204*	*20*
Rat # 8	0.4 ± 0.1	8.3 ± 3.4	25.0	38.5 ± 0.1	2.1 ± 0.4	25.1	32–206	20
*Rat # 9*	*3.3 ± 0.8*	*48.8 ± 7.7*	*25.3*	*37.3 ± 0.1*	*2.3 ± 0.2*	*25.2*	*138–188*	*20*
**Rat # 10**	**0.5 ± 0.1**	**12.2 ± 2.0**	**24.3**	**36.8 ± 0.1**	**1.8 ± 0.1**	**24.4**	**108–358**	**4**
**Rat # 11**	**6.0 ± 1.3**	**53.5 ± 5.2**	**25.1**	**37.6 ± 0.2**	**2.4 ± 0.4**	**25.1**	**128–572**	**0**
*Rat # 12*	*1.3 ± 0.2*	*23.5 ± 6.1*	*25.2*	*37.4 ± 0.1*	*1.8 ± 0.2*	*25.2*	*158–354*	*16*
Rat # 13	0.4 ± 0.1	8.9 ± 1.1	25.1	37.1 ± 0.1	1.6 ± 0.2	25.1	97–707	20
*Rat # 14*	*0.7 ± 0.1*	*15.6 ± 1.3*	*25.2*	*37.3 ± 0.1*	*2.1 ± 0.2*	*25.1*	*144–238*	*20*
**Rat # 15**	**0.9 ± 0.2**	**15.1 ± 1.4**	**24.6**	**37.5 ± 0.1**	**2.1 ± 0.2**	**24.5**	**118–566**	**8**
*Rat # 16*	*1.2 ± 0.3*	*24.7 ± 4.0*	*25.1*	*37.6 ± 0.1*	*2.3 ± 0.6*	*25.2*	*155–278*	*16*
**Rat # 17**	**0.6 ± 0.1**	**14.8 ± 3.1**	**24.3**	**37.2 ± 0.1**	**1.5 ± 0.3**	**24.2**	**26–113**	**8**
Rat # 18	1.1 ± 0.6	15.9 ± 2.8	25.0	37.4 ± 0.1	2.0 ± 0.5	25.0	20–423	16
Rat # 19	0.7 ± 0.1	15.8 ± 2.5	24.5	37.7 ± 0.1	2.0 ± 0.2	24.4	20–104	16
Rat # 20	1.0 ± 0.2	16.1 ± 1.3	24.5	37.6 ± 0.1	1.9 ± 0.4	24.5	21–120	16
**Rat # 21**	**1.1 ± 0.1**	**20.7 ± 1.9**	**25.4**	**37.4 ± 0.1**	**2.1 ± 0.2**	**25.4**	**27–295**	**4**
Rat # 22	0.7 ± 0.1	13.7 ± 1.5	25.1	37.4 ± 0.1	1.9 ± 0.2	25.1	34–559	20

Plasma melatonin levels showed marked variations among individuals in the peak levels and in the night-to-day ratio (Table 1). Interestingly, in six rats the night-to-day ratio was less than 2, whereas in the rat that showed arrhythmicity in R_w _and T_b _melatonin levels were high and rhythmic with a large night-to-day ratio (Table 1). In seven animals, serum melatonin levels peaked during the subjective day (from CT0 to CT8), thus suggesting that in these animals the circadian rhythm of serum melatonin desynchronized from the circadian rhythms of R_w _and T_b _(Table 1). No significant correlation was observed between the amplitude (or the levels) of the melatonin profile and the amplitude and mesor of the R_w _and T_b _rhythms (P > 0.1).

To further investigate the relationships among the R_w_, T_b _and melatonin rhythms, animals were divided into three different groups: 1) animals (N = 8) in which the rhythm of serum melatonin was synchronized with R_w _and T_b _rhythms; 2) animals (N = 6) in which the serum melatonin profile was synchronized with the R_w _and T_b _rhythms but had a reduced (less than 2-fold) amplitude; and 3) animals (N = 7) in which the serum melatonin rhythm was desynchronized from R_w _and T_b _rhythms.

Figure 1A-C shows representative records of R_w_, T_b _and melatonin levels obtained in a rat (#1 in Table 1) that did not show desynchronization or a reduced night-to-day serum melatonin ratio. Although melatonin levels in the animals belonging to this group were quite variable, all the animals showed a high night-to-day ratio (Table 1). The mean values of the circadian parameters of R_w _and T_b _for this group of animals are shown in Table 2.

**Table 2:** Circadian parameters for R_w _and T_b _(mean ± SEM). Group 1 = synchronized animals in which serum melatonin showed a high (more than 5) night-to-day ratio. Group 2 = synchronized animals in which serum melatonin showed a night-to-day ratio smaller than 2. Group 3 = desynchronized animals (i.e., animals in which the serum melatonin levels peaked during the subjective day). No significant differences were observed among the groups in any of the circadian parameters investigated (ANOVA, P > 0.1).

		Running wheel activity			Body Temperature		
	N	Mesor	Amplitude	τ	Mesor	Amplitude	τ
Group 1	8	0.9 ± 0.2	17.2 ± 3.4	24.9 ± 0.1	37.5 ± 0.1	1.9 ± 0.1	24.9 ± 0.1
Group 2	6	2.7 ± 0.9	31.5 ± 5.4	25.0 ± 0.1	37.3 ± 0.1	2.2 ± 0.2	25.0 ± 0.1
Group 3	7	1.4 ± 0.8	20.2 ± 5.9	24.8 ± 0.2	37.2 ± 0.1	1.9 ± 0.1	24.7 ± 0.2

Figure 1D-F shows two representative actograms for R_w _and T_b _rhythms and the melatonin profile for one animal (# 5 in Table 1) in which the amplitude of the melatonin rhythm was damped (i.e., less than 2 fold). In this group, peak melatonin levels showed less variability (range: 188–354 pg/ml) and the peak levels were lower that those recorded in the previous group (Table 1). Although in these animals the amplitude of the melatonin rhythm was reduced, the free-running period, the amplitude, and the mesor of the R_W _and T_b _rhythms were not different from those observed in the previous group of animals (t-test, P > 0.1 in all cases, Table 2).

Finally, Figure 1G-I shows the records of an animal (# 21) belonging to the group in which desynchronization from the R_W _and T_b _rhythms was observed. Though these animals had a melatonin profile that was desynchronized from the R_W _and T_b _rhythms, melatonin levels showed a marked variation over the 24 h and the peak values were not different from what was observed in the animals that did not desynchronize (t-test, P > 0.5, Table 1). Although in these animals the melatonin rhythms was desynchronized from the R_W _and T_b _rhythms, we did not observe any significant change in τ, amplitude or mesor of the RW and T_b _rhythms (t-test, P > 0.1 in all cases, Table 2).

## Discussion

The relationship between the circadian rhythms of locomotor activity and body temperature has been investigated in few studies. In humans, it has been reported that the body temperature rhythm is phase-advanced with respect to the activity rhythm [16] and, occasionally, these two rhythms may desynchronize [7,8]. However, studies in other mammalian species failed to observe this phenomenon either in nocturnal or in diurnal mammals [17,18], thus suggesting that in these animals the circadian rhythms of locomotor activity and body temperature are tightly coupled and, most likely, are controlled by a single circadian pacemaker [1,18]. The data obtained in this study support this view because they indicate that the R_w _and T_b _rhythms in Wistar rats are tightly coupled. Although we did not take special precautions to prevent masking of the T_b _rhythm by the R_w _rhythm, we observed no desynchronization between the R_w _and T_b _rhythms.

Our results also indicate that long term exposure to LL_dim _can induce desynchronization of the circadian rhythm of serum melatonin, and the amplitude of the circadian rhythm in serum melatonin may be dramatically reduced. Moreover, the observation that melatonin remained rhythmic in an animal in which R_w _and T_b _were arrhythmic further suggests that the regulation of melatonin rhythmicity is independent from the regulation of the running wheel activity and body temperature rhythms. These results confirm and expand our recent study [15] by showing that alteration in some parameters of the melatonin rhythm (i.e., desynchronization and amplitude) had no effects on τ, amplitude and mesor of the R_w _and T_b _rhythms. Such a result was unexpected because it is believed that melatonin plays an important role in the regulation of the circadian timing system as well as of body temperature [12,14].

Recent experimental evidence suggests that the SCN may contain several circadian pacemakers. For example, the circadian rhythm of arginine vasopressin and vasoactive intestinal polypeptide release in cultured SCN is regulated by different populations that can desynchronize from each other [19,20]. Spontaneous splitting of the locomotor activity rhythm under constant bright light may be the consequence of desynchronization of populations between the left and right SCN [21]. A very recent study using a forced desynchronization protocol has indicated the presence of two oscillators in the anatomically SCN subdivisions [22], thus suggesting that the SCN is composed of different populations of circadian oscillators that constitute regional pacemakers controlling specific circadian outputs.

In mammals the pineal gland is the major source of circulating melatonin, and several studies have shown that melatonin synthesis is under the control of a circadian pacemaker located in the SCN via a multisynaptic pathway [23]. Our study suggests that the circadian pacemaker driving melatonin synthesis is rather independent from the circadian pacemaker(s) driving the locomotor activity and the body temperature rhythms since it can desynchronize or damp without affecting these rhythms and, at the same time, it can remain rhythmic even in the case when R_w _and T_b _rhythms may became arrhythmic.

Remarkably, the reduced amplitude of the melatonin rhythm observed in several animals (Table 1 and Figure 1F) was caused by a clear increase of the basal melatonin levels and a decrease of peak levels. Such a result is well in agreement with our previous study in which we reported that pineal Arylalkylamine *N*-acetyltransferase mRNA levels are reduced in animal exposed to LL_dim _[15] and suggests that, in some animals, the signal by which the SCN drives the circadian rhythm of pineal melatonin synthesis may be reduced under long-term exposure to constant conditions. However, it must be also mentioned that this reduction in the amplitude of the serum melatonin rhythms may be due to the fact that peak and trough levels were missed due to the limited number of sampling points used.

In conclusion, the data presented in this study support the idea that the mammalian SCN is composed of a network of circadian pacemakers that control specific outputs, so that under specific experimental conditions (i.e., exposure to constant dim light or forced desynchrony protocols) these pacemakers may desynchronize. Our data also support the notion that in the rat the circadian rhythms of locomotor activity and body temperature are controlled by a single pacemaker.

## Competing interests

The author(s) declare that they have no competing interest.

## Authors' contributions

JA and NMB participated in data collection and data analysis. JA drafted the manuscript. GT directed the study and wrote the final version of the manuscript. All authors read and approved the final version of the article.
